# Crystal Facet Engineering of TiO_2_ Nanostructures for Enhancing Photoelectrochemical Water Splitting with BiVO_4_ Nanodots

**DOI:** 10.1007/s40820-022-00795-8

**Published:** 2022-01-25

**Authors:** Mi Gyoung Lee, Jin Wook Yang, Hoonkee Park, Cheon Woo Moon, Dinsefa M. Andoshe, Jongseong Park, Chang-Ki Moon, Tae Hyung Lee, Kyoung Soon Choi, Woo Seok Cheon, Jang-Joo Kim, Ho Won Jang

**Affiliations:** 1 Department of Materials Science and Engineering, Research Institute of Advanced Materials, Seoul National University, Seoul, 08826 Republic of Korea; 2 Department of Chemistry and Nanoscience, Ewha Womans University, Seoul, 03693 Republic of Korea; 3 National Research Facilities and Equipment Center, Korea Basic Science Institute, Daejeon, 34133 Republic of Korea; 4 Advanced Institute of Convergence Technology, Seoul National University, Suwon, 16229 Republic of Korea

**Keywords:** Crystal facet control, Bismuth vanadate, Titanium dioxide, Heterojunction, Water splitting

## Abstract

Although bismuth vanadate (BiVO_4_) has been promising as photoanode material for photoelectrochemical water splitting, its charge recombination issue by short charge diffusion length has led to various studies about heterostructure photoanodes. As a hole blocking layer of BiVO_4_, titanium dioxide (TiO_2_) has been considered unsuitable because of its relatively positive valence band edge and low electrical conductivity. Herein, a crystal facet engineering of TiO_2_ nanostructures is proposed to control band structures for the hole blocking layer of BiVO_4_ nanodots. We design two types of TiO_2_ nanostructures, which are nanorods (NRs) and nanoflowers (NFs) with different (001) and (110) crystal facets, respectively, and fabricate BiVO_4_/TiO_2_ heterostructure photoanodes. The BiVO_4_/TiO_2_ NFs showed 4.8 times higher photocurrent density than the BiVO_4_/TiO_2_ NRs. Transient decay time analysis and time-resolved photoluminescence reveal the enhancement is attributed to the reduced charge recombination, which is originated from the formation of type II band alignment between BiVO_4_ nanodots and TiO_2_ NFs. This work provides not only new insights into the interplay between crystal facets and band structures but also important steps for the design of highly efficient photoelectrodes.

**Supplementary Information:**

The online version contains supplementary material available at 10.1007/s40820-022-00795-8.

## Highlights

- Two types of BiVO_4_/TiO_2_ heterostructure photoanodes comprising TiO_2_ nanorods (NRs) and TiO_2_ nanoflowers (NFs) with different (001) and (110) crystal facets, respectively, were designed.
- The higher photoactivity of BiVO_4_/TiO_2_ NFs than BiVO_4_/TiO_2_ NRs was attributed to the improvement of charge separation by the TiO_2_ NFs.
- The formation of type II band alignment between BiVO_4_ nanodots and TiO_2_ NFs expedited electron transport and reduced charge recombination.

**Supplementary Information:**

The online version contains supplementary material available at 10.1007/s40820-022-00795-8.

## Graphical abstract

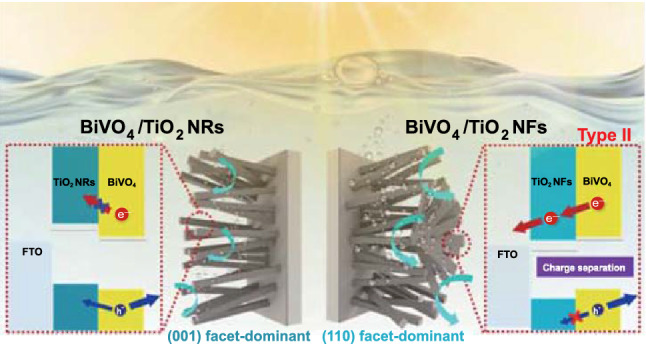

## Introduction

Solar-driven photoelectrochemical (PEC) water splitting is considered a major breakthrough to settle an energy crisis. Despite its promise, the commercialization of PEC water splitting has been limited because of the relatively low efficiency and stability [1]. Although metal oxide-based photoelectrodes such as TiO_2_, Fe_2_O_3_, WO_3_, and BiVO_4_ can be easily synthesized, major limitations such as charge recombination and short carrier lifetimes of from picosecond (*ps*) to nanosecond (*ns*) restrict their photoactivity.

The construction of heterostructure photoelectrodes is an effective approach to improve light absorption, carrier separation, and charge transfer efficiencies [2, 3]. Since it relies on the appropriate band alignment like type II heterojunction, the combination of photoelectrode materials having proper band edge positions is a key for determining their functionality in PEC water splitting. Among various heterostructures such as Fe_2_O_3_/WO_3_ [4], BiVO_4_/WO_3_ [5–7], Fe_2_O_3_/SnO_2_ [8–10], In_2_S_3_/In_2_O_3_ [11], and BiVO_4_/SnO_2_ [12, 13], BiVO_4_-based heterostructure photoanodes have been widely studied since short charge diffusion lengths and lifetimes of BiVO_4_ cause severe charge recombination. In a representative BiVO_4_/WO_3_ heterostructure, WO_3_ enhances the charge separation as a hole blocking layer of BiVO_4_, forming the type II band alignment. However, it has a relatively positive flat band potential of about 0.4 V versus (vs.) a reversible hydrogen electrode (RHE), which results in potential energy losses for electrons as they are transferred from the WO_3_ to the BiVO_4_, limiting the photovoltage of the combined system [5–7]. Also, the oxidation of surface hydroxyl groups of WO_3_ causes the formation of peroxo-species, which reduces the stability of heterostructure photoanodes [13].

Compared to the WO_3_, rutile TiO_2_ is highly stable in a wide range of pH and has a relatively negative flat band potential of about 0.2 V versus RHE, which does not significantly limit the photovoltage obtainable from BiVO_4_ [14–16]. However, TiO_2_ has an intrinsic limitation of low electrical conductivity to be used as the hole blocking layer for BiVO_4_ [17]. To resolve the problem, the increment of carrier concentration through doping and nanostructuring is required. In particular, both high surface area and electrical conductivity can be obtained by designing one-dimensional (1D) nanostructures such as nanorods (NRs), nanowires, and nanoflowers (NFs). Andoshe et al*.* developed S, N co-doped TiO_2_ NRs and increased their carrier concentration and electrical conductivity [18]. The second problem is a mismatched band structure between BiVO_4_ and TiO_2_. A relatively positive valence band edge of TiO_2_ makes it difficult to form the type II heterostructure suitable for charge separation. In order to utilize TiO_2_ as the hole blocking layer for BiVO_4_, the control of band edge position is necessary, and crystal facet engineering has emerged as an emerging strategy. According to the previous works, metal oxides provided various band edge positions depending on their crystal facets [19–21]. For instance, Wang et al. demonstrated that the (111) facet of Cu_2_O, with a lower work function than the (100) facet, is unfavorable for the migration of holes from the Cu_2_O surface to Pd through a semiconductor–metal junction [22]. It is reasonable to select and control the contact facet for the heterostructure to achieve suitable band structures and efficient charge separation.

In this work, we compare two types of BiVO_4_/TiO_2_ heterostructure photoanodes with different (001) and (110) crystal facets of TiO_2_, which are NRs and NFs, respectively. After BiVO_4_ nanodots are conformally electrodeposited on the surface of the TiO_2_ nanostructures, the photocurrent density of BiVO_4_/TiO_2_ NFs increases by about 4.7 times, while that of BiVO_4_/TiO_2_ NRs decreases. Based on the analysis of charge carrier dynamics, it is revealed that the difference is derived by the charge separation ability of crystal facet-engineered TiO_2_. The ultraviolet photoemission spectroscopy (UPS) represents band edge positions of TiO_2_ nanostructures are significantly dependent on their crystal facet. Since TiO_2_ NFs with (110) facets have relatively negative band edges, as a hole blocking layer they form type II heterojunction with BiVO_4_ nanodots. In addition, the initial facet of TiO_2_ has a decisive effect on the final architecture of the BiVO_4_/TiO_2_ due to the difference in the tendency of the BiVO_4_ electrodeposition on the nanostructured TiO_2_. In a word, crystal facet engineering plays a key role in affecting charge separation and in determining photoactivity. These findings provide a feasible avenue to adjust diverse metal oxides for use as photoelectrodes for desirable solar water splitting.

## Experimental Section

### Synthesis of TiO_2_ NRs and TiO_2_ NFs

TiO_2_ NRs and TiO_2_ NFs were grown on fluorine doped tin oxide (FTO) glass substrates using hydrothermal synthesis similar to those in a previously reported paper [18]. Briefly, the precursor solution was prepared with 0.8 mL of titanium (IV) butoxide (C_16_H_36_O_4_Ti, 97%, Aldrich), 5 mg of sulfamic acid (NH_2_SO_3_H, 99.3%, Aldrich), 25 mL of HCl (38%, Daejung), and 25 mL of deionized water (dH_2_O) under magnetic stirring. After vigorous magnetic stirring for another 10 min, the solution was poured into Teflon vessel. The Teflon vessel, which contained the precursor solution and the FTO glass, was inserted into the autoclave and heated for 4 h at 180 °C in an oven and kept inside until the temperature reached room temperature. The synthesized TiO_2_ NRs were removed from the Teflon vessel and rinsed repeatedly using dH_2_O. After sufficient rinsing, the TiO_2_ NRs were annealed at 500 °C for 3 h with 5% H_2_/95% N_2_ gas. For the synthesis of the TiO_2_ NFs, a larger amount of sulfamic acid was added to the precursor solution, and the reaction time was increased over that of the TiO_2_ NRs. A precursor solution for the growth of TiO_2_ NFs was prepared with 100 mg of sulfamic acid (NH_2_SO_3_H, 99.3%, Aldrich), 0.8 mL of titanium butoxide (C_16_H_36_O_4_Ti, 97%, Aldrich), and 50 mL of HCl solution (25 mL of dH_2_O and 25 mL of concentrated HCl (38%, Daejung)). After vigorous stirring until the solution became completely clear, the solution was transferred to the Teflon vessel and moved into an autoclave. The autoclave was sealed and heated to the reaction temperature (180 °C) in an oven for 12 h and maintained until the autoclave was cooled to room temperature. The synthesized TiO_2_ NFs were washed with dH_2_O and annealed at 500 °C for 3 h under 5% H_2_/95% N_2_ gas.

### Fabrication of BiVO_4_/TiO_2_ Heterostructure Photoanodes

BiVO_4_ was electrodeposited onto the two types of TiO_2_ nanostructures using previously reported methods [5, 23]. A precursor was prepared by dissolving bismuth nitrate pentahydrate (BiN_3_O_9_, 98%, Junsei) in a solution of vanadium oxide sulfate hydrate (VOSO_4_, 99.99%, Aldrich) at pH < 0.5 with nitric acid (HNO_3_, 67%, Junsei). Then, sodium acetate (CH_3_COONa, 99%, Aldrich) was added, raising the pH to ~ 5.1, which was then adjusted to pH 4.7 using a few drops of concentrated HNO_3_. This mildly acidic pH condition is necessary because, at pH values > 5, vanadium (IV) precipitates form in the solution. Pulsed anodic electrodeposition was conducted in a standard three-electrode system with a working electrode of nanostructured TiO_2_, an Ag/AgCl reference electrode, and a platinum counter electrode. The electrodeposition was potentiostatically carried out at 1.95 V versus Ag/AgCl at 80 °C and annealed at 500 °C for 6 h in air at a heating rate of 2 °C per minute to crystallize.

### PEC Measurements

The photocurrent versus potential curves with a scan rate of 10 mV s^−1^ and photocurrent versus time curves were recorded with a solar simulator with an AM 1.5 G filter. The light intensity of the solar simulator was calibrated to 1 sun (100 mW cm^−2^). The incident photon-to-current conversion efficiency (IPCE) was measured at 1.23 V versus RHE. The electrochemical impedance spectroscopy (EIS) was conducted by applying 1.23 V versus RHE. The sweeping frequency ranged from 100 to 0.1 kHz, with an AC amplitude of 10 mV. The measured spectra were fitted using ZSimpWin software. The Mott-Schottky (M-S) plots were plotted by potential scan in AC condition with a frequency of 1 kHz with the light off. The Faradaic efficiency and gas evolution were calculated by the gas chromatography system (7890B, Agilent Technologies) in an air-tight cell at 1.23 V versus RHE.

### Characterization

The morphologies of all photoanodes were characterized by field-emission scanning electron microscopy (FESEM, MERLIN Compact, ZEISS). High-resolution transmission electron microscopic (HR-TEM) images and elemental distributions were obtained with TEM (JEM-2100F, JEOL) equipped energy-dispersive spectroscope (EDS). X-ray diffraction (XRD, D8-Advance, Bruker) characterization was performed to confirm the crystalline phase of the TiO_2_ NRs, TiO_2_ NFs, and BiVO_4_/TiO_2_ NFs. The absorption spectra of the TiO_2_ NRs, TiO_2_ NFs, and BiVO_4_ were measured by UV–Visible spectroscopy (V-770, JASCO). The band structures of the BiVO_4_/TiO_2_ NFs and BiVO_4_/TiO_2_ NRs were determined by analyzing the UPS (Ultra DLD) in advanced in-situ surface analysis system (AiSAS)). The time-resolved photoluminescence of TiO_2_ NFs, TiO_2_ NRs, BiVO_4_/TiO_2_ NFs, and BiVO_4_/TiO_2_ NRs was measured using photoluminescence (PL, FlouTime 300, PicoQuant) with an excitation laser having a wavelength of 405 nm.

### FDTD Simulation

The simulations were performed on the FDTD solutions program (Lumerical Solutions). The dielectric function of rutile TiO_2_ was adopted from a prominent report [24], and FTO was considered as a perfect dielectric with a constant refractive index of 2.2 based on the analysis of previous literature [25]. The detailed simulation condition is displayed in Fig. S2. The mesh size was 5 × 5 nm^2^.

## Results and Discussion

### Synthesis and FDTD Simulations of TiO_2_ Nanostructures

**Fig. 1:**
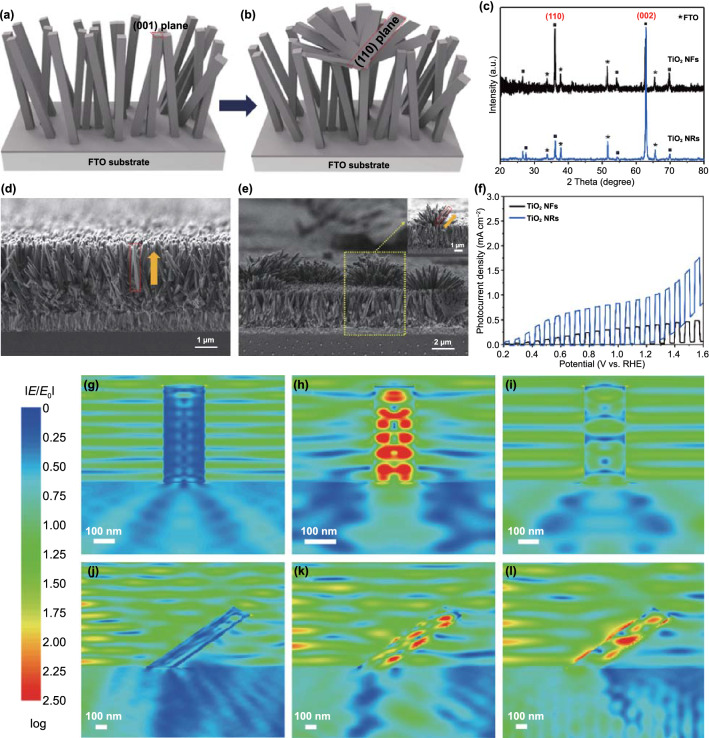
Schematic illustration of **a** TiO_2_ NRs and **b** TiO_2_ NFs growth via hydrothermal synthesis. **c** XRD patterns of TiO_2_ NRs and TiO_2_ NFs. Cross-sectional SEM images of **d** TiO_2_ NRs and **e** TiO_2_ NFs. **f** *J*–*V* curves of TiO_2_ NRs and TiO_2_ NFs in 0.5M potassium phosphate (K–P_i_) buffer with 1M Na_2_SO_3_. FDTD simulations of near field enhancement of TiO_2_ NRs on FTO under **g** 350 nm, **h** 450 nm, and **i** 550 nm photons. FDTD simulations of near field enhancement of TiO_2_ NFs on FTO under **j** 350 nm, **k** 450 nm, and **l** 550 nm photons

To synthesize BiVO_4_/TiO_2_ nanostructures, we introduced an all-solution process comprising hydrothermal synthesis and electrodeposition, which is cost-effective and eco-friendly fabrication method for photoelectrode [26]. First, two types of TiO_2_ nanostructures, which are TiO_2_ NRs and TiO_2_ NFs, were prepared by the one-step hydrothermal synthesis method. By controlling the reaction time, precursor concentration, and pH, the morphology and alignment of TiO_2_ nanostructures were changed into NRs and NFs, as illustrated in Fig. 1a, b. TiO_2_ NRs and NFs have rutile structures with different lattice plane intensities, i.e., (001) and (110), as shown in XRD analysis (Fig. 1c). In the case of TiO_2_ NRs, TiO_2_ preferentially grew to the (001) direction and it makes the vertically aligned 1D structures, as shown in Fig. 1d, whereas TiO_2_ NFs showing the increased (110) direction peak in XRD have a tendency to grow laterally, as shown in Fig. 1e. The long reaction time of 10 h not only caused new heterogeneous nucleation at the tip of the nanorods, but also led to the growth of nanoflowers. As a result, the morphology of TiO_2_ NFs formed on the TiO_2_ NRs was implemented. Also, in the hydrothermal synthesis of TiO_2_, acids, such as hydrochloric acid, sulfuric acid, sulfonic acid, and sulfamic acid, play highly important roles for the preferential growth as well as stabilization of the rutile phases [27]. We made a more acidic environment by adding sufficient sulfamic acid in order to induce the specific facet growth of nanoflowers. Based on the long reaction time and acidic environment, unlike previous reports for synthesizing the TiO_2_ NFs, we enable to construct well-maintained rectangular shape of TiO_2_ NFs without additional process [28, 29].

To compare the photoactivity according to the major facet of TiO_2_ nanostructures, photocurrent density and IPCE of the TiO_2_ NRs and NFs are measured, as shown in Figs. 1f and S1. Both photocurrent density and IPCE of the TiO_2_ NRs were higher than those of the TiO_2_ NFs. Since the (001) facet possesses the highest surface energy due to the coordinately unsaturated Ti and O atoms on (001) and very large Ti–O–Ti bond angles, the (001) facet of TiO_2_ was more reactive than the other facets such as (110) and (101) [30]. And vertically aligned 1D TiO_2_ NRs can make direct and ordered channels for electron transport and enhance light absorption. Furthermore, minority charge diffusion paths can be decoupled to different directions to enhance the photocharge collection efficiency [31].

To clearly understand light-harvesting capacity according to the TiO_2_ crystal facets, we performed a finite-domain time-difference (FDTD) simulation, as shown in Fig. 1g–i. The electric field (|*E*|) distribution was calculated and visualized using the FDTD method to assist in understanding the light absorption mechanism. The model for the FDTD simulation is shown in Fig. S2. We considered vertical-, 30°-tilted, and 50°-tilted TiO_2_ NRs by reflecting the SEM images (Fig. 1d, e). The results of the simulated absorption distribution along the z-axis acquired at light wavelengths of 350, 450, and 550 nm. The FDTD results clearly show strong absorption enhancement in the (001)-facet-dominant TiO_2_ NRs compared to the (110)-facet-dominant TiO_2_ NFs, especially at the wavelength of 450 nm, indicating that the (001) facet traps the incident light efficiently. As shown in Figs. S3, S4, the real configuration of TiO_2_ NFs showed a similar tendency with a piece of TiO_2_. These FDTD simulation results are closely related to the photoactivity of facet-controlled TiO_2_ nanostructures in linear sweep voltammetry (LSV) (Fig. 1f).

### Characterization and PEC performances of TiO_2_ NRs and BiVO_4_/TiO_2_ NRs

**Fig. 2:**
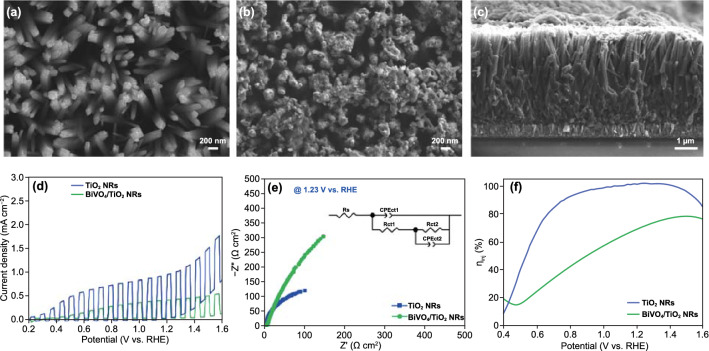
Top SEM images of **a** TiO_2_ NRs and **b** BiVO_4_/TiO_2_ NRs. **c** Cross-sectional SEM image of BiVO_4_/TiO_2_ NRs. **d** LSV for TiO_2_ NRs and BiVO_4_/TiO_2_ NRs in 0.5M K–Pi buffer with 1M Na_2_SO_3_. **e** Electrochemical impedance spectra (EIS) of TiO_2_ NRs and BiVO_4_/TiO_2_ NRs at 1.23 V (vs. RHE) in 0.5M K– Pi buffer with 1M Na_2_SO_3_. Inset shows equivalent circuit. **f** Charge injection efficiency of TiO_2_ NRs and BiVO_4_/TiO_2_ NRs

Controlled facets affect not only the photocurrent density of TiO_2_ nanostructures but also the electrodeposition trend of BiVO_4_ on the TiO_2_. First, we constructed heterostructure photoanodes of BiVO_4_/TiO_2_ NRs to demonstrate the effect of major facets of TiO_2_ on the final architecture of BiVO_4_/TiO_2_ nanostructures and the following photoactivities. On the TiO_2_ NRs, electrodeposited BiVO_4_ nanodots agglomerate, and thus the tops of the BiVO_4_/TiO_2_ NRs are close to each other, as shown in Figs. 2a–c and S5. It is related to the concentration gradient in the solution under the electrodeposition process since ions tend to converge toward the vertex of TiO_2_ NRs due to high conductivity. Agglomerated BiVO_4_ nanodots block the gaps between the TiO_2_ NRs, which might be deteriorating the light absorption and electrolyte permeation for PEC water splitting. As a result, the photocurrent density of the BiVO_4_/TiO_2_ NRs (0.35 mA cm^−2^ at 1.23 V vs. RHE) was significantly less than that of pristine TiO_2_ NRs (0.91 mA cm^−2^ at 1.23 V vs. RHE), as shown in Figs. 2d and S6. Also, as shown in Fig. S7, the IPCE of BiVO_4_/TiO_2_ NRs was significantly decreased to 22% under the wavelength of 390 nm at 1.23 V versus RHE, which means there are lots of charge recombination after the introduction of BiVO_4_ on TiO_2_ NRs.

We also analyzed the EIS to compare the charge transfer kinetics between the BiVO_4_/TiO_2_ NRs and TiO_2_ NRs, as shown in Fig. 2e. Considering our architecture, we used the Haman equivalent circuit composed of three resistances and two capacitances since the EIS circuit is closely related to the structure of photoelectrodes [23]. In the EIS circuit, *R*_s_ is the series resistance, *C*_s_ and *C*_ct_ are the constant phase elements (CPE) for the semiconductor interface and the electrolyte/electrode interface, respectively, and *R*_ct1_ and *R*_ct2_ are the charge transfer resistances across the semiconductor interface and electrode/electrolyte interface, respectively. The values of *R*_s_, *R*_ct1_, and *R*_ct2_ obtained from the fittings are summarized in Table S1. The high photoactivity is represented by a small semicircle in the Nyquist plot. After the deposition of BiVO_4_ nanodots, the charge transfer resistance across the electrode/electrolyte interface increased from 259.89 to 1948.06 Ω cm^2^, showing the larger semicircles of BiVO_4_/TiO_2_ NRs compared to that of TiO_2_ NRs. As shown in SEM images of BiVO_4_/TiO_2_ NRs (Fig. 2b, c), the agglomerated BiVO_4_ reduced the porosity of the TiO_2_ NRs, which is a structure interfering with the electrolyte permeation. Therefore, bulky BiVO_4_ and reduced porosity of TiO_2_ NRs become the main factors that increase the charge transfer resistance of BiVO_4_/TiO_2_ NRs photoanode. This result was also represented in that BiVO_4_/TiO_2_ NRs have a lower charge injection efficiency than TiO_2_ NRs (Fig. 2f), which led to the deterioration of the PEC performances.

### Characterization and PEC Performances of TiO_2_ NFs and BiVO_4_/TiO_2_ NFs

**Fig. 3:**
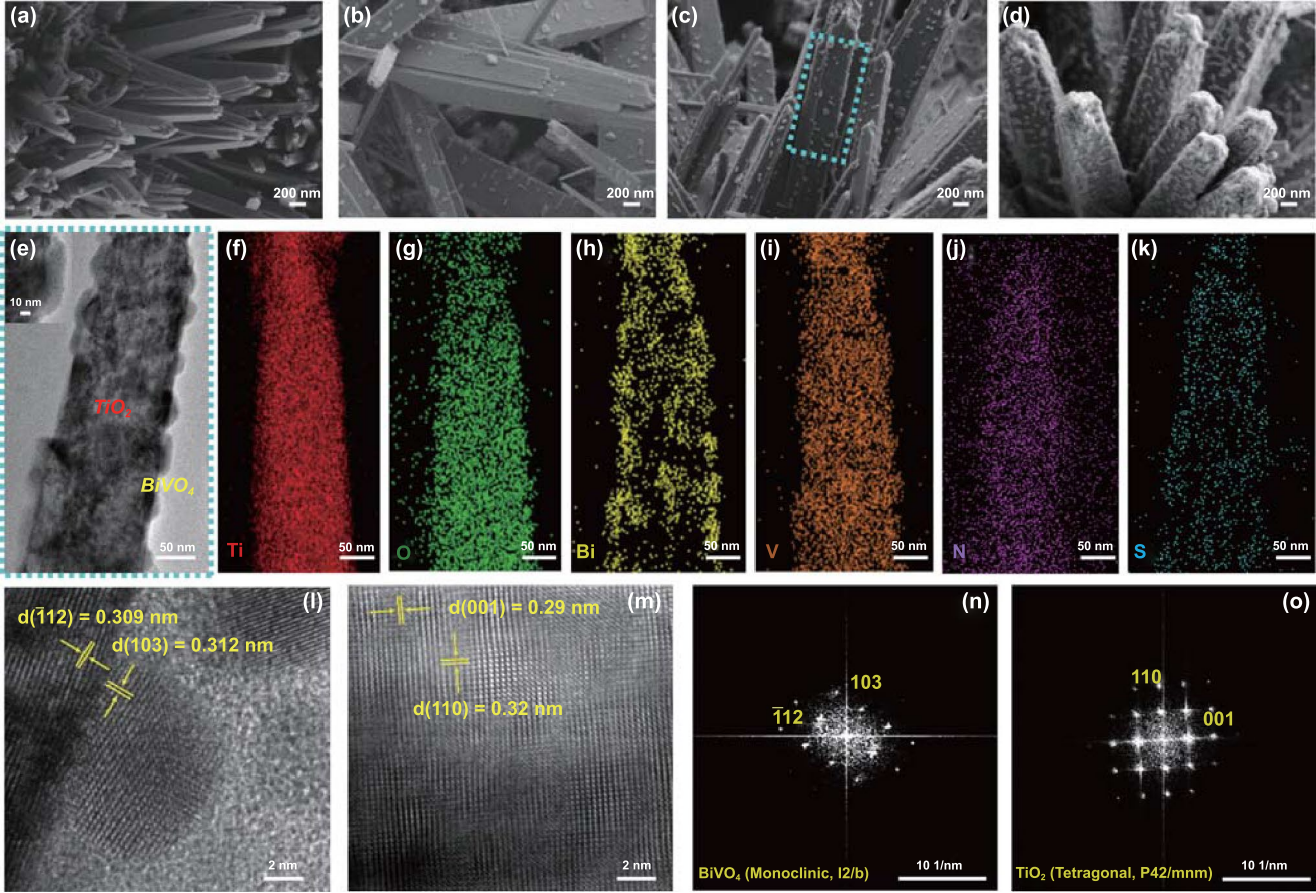
SEM images of **a** TiO_2_ NFs and BiVO_4_/TiO_2_ NFs with different deposition cycles of BiVO_4_: **b** 6 cycles, **c** 18 cycles, and **d** 36 cycles. **e** TEM image of BiVO_4_/TiO_2_ NFs deposited by 18 cycles. Inset shows HR-TEM image of BiVO_4_/TiO_2_ NFs. EDS mapping images of **f** Ti, **g** O, **h** Bi, **i** V, **j** N, and **k** S. **l** Crystalline planes of (− 112) and (103) of BiVO_4_, **m** Crystalline planes of (001) and (110) of TiO_2_ NFs. FFT patterns of **n** BiVO_4_ and **o** TiO_2_ NFs

In order to investigate differences in nanostructures and photoactivities according to the crystal facet, we set up TiO_2_ NFs with (110) facets as a bottom layer for BiVO_4_ nanodots. Unlike on the TiO_2_ NRs, the conformal coating of extremely thin BiVO_4_ nanodots across the entire surface of the TiO_2_ NFs could be possible through the control of pulse cycles during the electrodeposition, as shown in Fig. S8. According to SEM images in Fig. 3a–d, the surfaces of TiO_2_ NFs were totally covered with BiVO_4_ nanodots by increasing the pulse cycles of BiVO_4_. A cross-sectional SEM image in Fig. S9 also showed conformally decorated BiVO_4_ on TiO_2_ NFs. Through the TEM equipped with EDS, we confirmed the detailed surface morphologies and elemental distributions of BiVO_4_/TiO_2_ NFs. The TEM image in NFs region (Fig. 3e) also indicated that BiVO_4_ nanodots conformally covered on the surface of the TiO_2_ NFs. The inset shows an enlarged TEM image of BiVO_4_ nanodots. As shown in EDS mappings (Fig. 3f–k), all the constituent elements were well represented. High-resolution TEM (HR-TEM) images and a fast Fourier transform (FFT) pattern are shown in Fig. 3l–o. According to high-resolution TEM (HR-TEM) images and fast Fourier transform (FFT) patterns, electron diffraction patterns of the selected area showed *d*-spacing of 0.309 and 0.312 nm (Fig. 3l), corresponding to the (− 112) and (103) planes of monoclinic BiVO_4_, respectively (Fig. 3n), and *d*-spacing of 0.29 and 0.32 nm (Fig. 3m), which can be assigned to the (001) and (110) planes of rutile TiO_2_ NFs, respectively (Fig. 3o). These results are well-matched with the XRD analysis (Fig. S10).

**Fig. 4:**
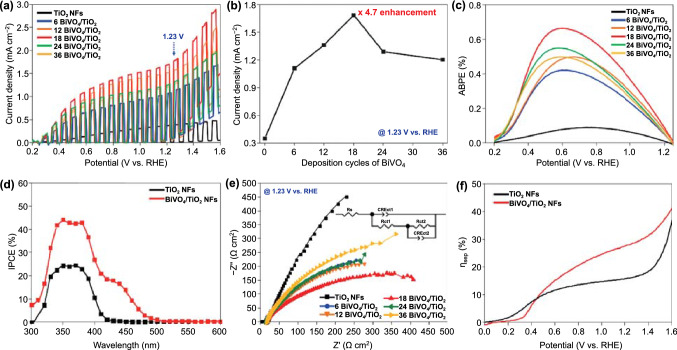
**a** LSV of TiO_2_ NFs and BiVO_4_/TiO_2_ NFs with different deposition cycles of BiVO_4_ in 0.5M K–P_i_ buffer with 1M Na_2_SO_3_. **b** Photocurrent density of BiVO_4_/TiO_2_ NFs with different deposition cycles of BiVO_4_ at 1.23 V (vs. RHE). **c** ABPE of TiO_2_ NFs and BiVO_4_/TiO_2_ NFs with different deposition cycles of BiVO_4_. **d** IPCE at 1.23 V (vs. RHE) of TiO_2_ NFs and 18 BiVO_4_/TiO_2_ NFs. **e** EIS of TiO_2_ NFs and BiVO_4_/TiO_2_ NFs with different deposition cycles of BiVO_4_ at 1.23 V (vs. RHE) in 0.5M K–P_i_ buffer with 1M Na_2_SO_3_. Inset shows equivalent circuit. **f** Charge separation efficiency of TiO_2_ NFs and 18 BiVO_4_/TiO_2_ NFs

PEC measurements of the BiVO_4_/TiO_2_ NFs with different coverages of BiVO_4_ were performed using a standard three-electrode cell with an electrolyte of 0.5M potassium phosphate (K–P_i_) buffer and 1M sodium sulfite (Na_2_SO_3_) at a scan rate of 10 mV s^−1^ under a AM 1.5G solar light. Oxidation of sulfite (SO_3_^2−^/SO_3_^−^, *E*° = 0.73 V vs. NHE; SO_3_^2¬^/S_2_O_6_^2¬^, E° = 0.026 V vs. NHE), is thermodynamically and kinetically much more favorable than water oxidation [32]. Therefore, surface recombination losses due to slow interfacial hole transfer kinetics can be assumed to be negligible for the photo-oxidation of sulfite. BiVO_4_/TiO_2_ NFs were fabricated by adjusting the electrodeposition cycles of BiVO_4_ from 0 to 36 (i.e., n BiVO_4_ = n cycles electrodeposited BiVO_4_), and their LSV curves were recorded under the chopped (on/off) light condition. As shown in Fig. 4a, the photocurrent densities of the BiVO_4_/TiO_2_ NFs gradually increase until the number of electrodeposition cycles increases to 18 cycles and then starts to decrease. As the increase in the deposition cycle of BiVO_4_, the density of BiVO_4_ nanodots covering the TiO_2_ NFs increases (Fig. S8b–d), adding the charge transfer sites. However, after the optimized cycles (18 cycles), the agglomeration of BiVO_4_ nanodots is observed at the tip of TiO_2_ NFs (Fig. S8e–f), interfering with the charge transfer. The highest photocurrent density of 1.7 mA cm^−2^ was obtained at 1.23 V versus RHE for the 18 BiVO_4_/TiO_2_ NFs. This value corresponds to 4.7 and 4.8 times that of the TiO_2_ NFs and BiVO_4_/TiO_2_ NRs, respectively. In addition to the enlarged absorption wavelength range from the ultraviolet (UV) to ultraviolet–visible (UV–Vis) region), improved charge separation is decisive for the cause of PEC performances of BiVO_4_/TiO_2_ NFs. The electron–hole recombination is structurally reduced because the conformally coated BiVO_4_ nanodots on TiO_2_ NFs enlarge the depletion layer and shorten the charge diffusion length to the interface [14, 33]. The small size of the BiVO_4_ nanodots allows for a high collection efficiency of electrons by the TiO_2_ NFs, and the proximity of the semiconductor liquid junction allows holes to reach the surface to perform the water splitting reaction [1, 14, 33, 34]. However, after the optimal deposition cycle, increasing the surface area also has negative effects such as the formation of surface defects and grain boundaries, which degrade PEC performances. In this respect, the design of photoanodes with appropriate active areas is crucial for photoactivity [5, 35, 36].

The PEC water splitting efficiency of photoelectrodes was also quantitatively evaluated using the applied bias photon-to-current conversion efficiency (ABPE) as follows:

1 $${\text{ABPE }}\left( \% \right) \, = J_{{{\text{ph}}}} \left( {{1}.{23 } - V_{{\text{b}}} } \right)*{1}00/P_{{{\text{total}}}}$$

where *V*_b_ is the applied bias (vs. RHE), *J*_ph_ is the photocurrent density at the measured potential, and *P*_total_ is the power density of light. The ABPE takes applied potential into account as important factor in the conversion of solar to chemical energy [37]. As shown in Fig. 4c, maximum photoconversion efficiency of 0.65% was achieved for the 18 BiVO_4_/TiO_2_ NFs at 0.61 V versus RHE, which was 8.1 times higher than that of the pristine TiO_2_ NFs. Also, the maximum photoconversion efficiencies for all BiVO_4_/TiO_2_ NFs were obtained at a lower applied potential than the pristine TiO_2_ NFs. These are associated with the reduction of the hole injection barrier at the interface between the TiO_2_ NFs and electrolyte by introduction of the BiVO_4_ nanodots [36]. According to the IPCE spectra (Fig. 4d), the absorption wavelength edge was expanded from 430 (UV region) to 512 nm (UV–Vis region) after the introduction of BiVO_4_. It also led to a 44% enlargement of maximum IPCE at a wavelength of 350 nm. In the chronoamperometry at 1.23 V versus RHE under 1 sun illumination, the 18 BiVO_4_/TiO_2_ NFs showed stable PEC performances for 6 h, as shown in Fig. S8h.

We also analyzed the EIS to figure out the charge transfer kinetics of BiVO_4_/TiO_2_ NFs, and the measured data were fitted to the equivalent circuit, as shown in Fig. 4e [23]. Unlike BiVO_4_/TiO_2_ NRs, the charge transfer resistance of all BiVO_4_/TiO_2_ NFs was drastically decreased compared to pristine TiO_2_ NFs. These results represent the effective charge transfer at the interfaces of each semiconductor and the semiconductor/electrolyte by suppressing the charge recombination. Of these, the lowest value of charge transfer resistances (*R*_ct1_ = 0.84 Ω cm^−2^ and *R*_ct2_ = 475.11 Ω cm^−2^) were recorded for the 18 BiVO_4_/TiO_2_ NFs, which well corresponds to the tendency of photocurrent density (Fig. 4b).

To gain more insight into the crystal facet effect, we investigated the electronic characteristics using Mott–Schottky (M-S) analysis to grasp a flat band potential by using Eq. (2):

2 $$C^{{ - {2}}} = \, \left( {{2}/{\text{e}}\varepsilon \varepsilon_{0} N_{{\text{d}}} } \right)\left[ {V - V_{FB} - k_{B} T/e} \right]$$

where *C* is the capacitance of the space charge layer, *e* is the electron charge (1.602 10^–19^ C), ε is the dielectric constant, *ε*_0_ is the permittivity of vacuum (8.854 10^–12^ F m^−1^), *V* (vs. RHE) is the applied potential, *V*_*FB*_ (vs. RHE) is the flat band potential, *k*_*B*_ is the Boltzmann constant (1.381 10^–23^ J K^−1^), T is the temperature (298 K), *E* is the applied potential, and N_d_ is the charge carrier (donor) density. The flat band potentials were determined from the intercepts of the 1/C^2^ versus *V* curve, subtracting *k*_*B*_*T/e* = 0.025 V from the intercept. And the carrier concentration can be calculated from the slope of the M-S curves. As the slope of the M-S plot flattens, the carrier concentration increases. The flat band potential of the TiO_2_ NFs is more cathodically shifted about 100 mV than the TiO_2_ NRs, as shown in Fig. S11. It is favorable for the electrons to pass through the circuit to the counter electrode [5]. We analyzed the M-S plot after electrodeposition of BiVO_4_ onto the TiO_2_ NFs and TiO_2_ NRs. As shown in Fig. S11a, the donor density of BiVO_4_/TiO_2_ NFs was increased, which means the internal resistance of BiVO_4_/TiO_2_ NFs is reduced, allowing the carrier to transport much faster than the pristine TiO_2_ NFs. On the other hand, as shown in Fig. S11b, the carrier concentration of the BiVO_4_/TiO_2_ NRs was drastically reduced, which derives the decrease in photoactivity. These results indicate that the facet and morphology control of the bottom layer could be a significant factor in controlling PEC water splitting kinetics.

We also measured charge separation efficiency (*η*_sep_) in the potential from 0 V to 1.6 V versus RHE. As shown in Fig. 4e, the BiVO_4_/TiO_2_ NFs exhibited 2.5 times higher *η*_sep_ than the pristine TiO_2_ NFs at 1.23 V versus RHE. It directly reveals that the enhanced photocurrent density of BiVO_4_/TiO_2_ NFs was mainly originated from the improved charge separation between BiVO_4_ nanodots and TiO_2_ NFs. To support this result, we also measured PEC water oxidation performances of TiO_2_ NFs and 18 BiVO_4_/TiO_2_ NFs in 0.5M K– P_i_ buffer without hole scavenger. As shown in Fig. S12a, BiVO_4_/TiO_2_ NFs showed more efficient water oxidation and higher photocurrent density compared to TiO_2_ NFs at 1.23 V versus RHE. The lower photocurrent density of BiVO_4_/TiO_2_ NFs in the lower potential region than that of TiO_2_ NFs was derived from the sluggish hole transfer of BiVO_4_. To expedite the hole transfer at the low potential, an additional introduction of oxygen evolution catalyst to BiVO_4_ is necessary [13]. Also, BiVO_4_/TiO_2_ NFs showed a 29% higher maximum IPCE and wider absorption wavelength range than TiO_2_ NFs as shown in Fig. S12b. We also measured the EIS of TiO_2_ NFs and 18 BiVO_4_/TiO_2_ NFs at 1.23 V versus RHE in K–Pi buffer electrolyte. As shown in Fig. S12c, BiVO_4_/TiO_2_ NFs showed a smaller semicircle compared to TiO_2_ NFs, which represented the smaller charge transfer resistance during the water oxidation. In the chronoamperometry at 1.23 V versus RHE in K–Pi buffer electrolyte, the 18 BiVO_4_/TiO_2_ NFs showed stable PEC performances for 2 h, as shown in Fig. S12d. By using gas chromatography, we measured gas evolution and Faradaic efficiency of 18 BiVO_4_/TiO_2_ NFs at 1.23 V versus RHE. As shown in Fig. S12e, the 18 BiVO_4_/TiO_2_ NFs photoanode and Pt cathode continuously generated oxygen and hydrogen gas. Near-complete Faradaic efficiency was achieved, representing that the photogenerated charge carriers were mostly used for evolving the oxygen and hydrogen gases.

### Studies of Charge Carrier Dynamics and Band Structures

**Fig. 5:**
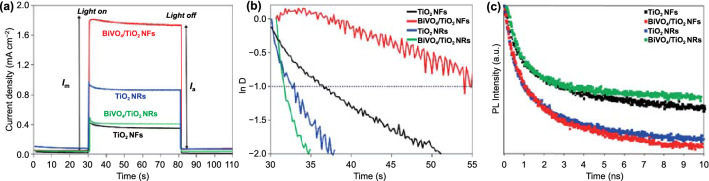
**a** The transient photocurrent decay that occurs immediately upon illumination at 1.23 V (vs. RHE). **b** Transient decay times for TiO_2_ NFs, TiO_2_ NRs, BiVO_4_/TiO_2_ NFs, and BiVO_4_/TiO_2_ NRs. **c** TRPL of TiO_2_ NFs, TiO_2_ NRs, BiVO_4_/TiO_2_ NFs and BiVO_4_/TiO_2_ NRs

To profoundly understand the effect in the crystal facet of TiO_2_ nanostructures to the charge transport and recombination behavior, the transient photocurrent decay occurring immediately upon illumination was evaluated. As shown in the chronoamperometry at 1.23 V versus RHE (Fig. 5a), when the light was switched on, a photocurrent spike was observed due to the rapid generation of electron and hole pairs. It would lead to severe charge recombination and cause the decrease in the photoactivity for the water splitting reaction. In general, the charge recombination can be caused by the accumulation of either electrons in the bulk or holes at the surface. The accumulation of holes would cause an equally large cathodic transient when the light is switched off, and electrons in the conduction band react with the accumulated hole. However, cathodic transients can scarcely be observed in Fig. 5a, suggesting the accumulation of holes at the surface of the films is not the main recombination process in both BiVO_4_/TiO_2_ NFs and BiVO_4_/TiO_2_ NRs. Thus, it is represent that the transient decays were mainly dependent to the accumulation of electrons due to the poor electron transport in the photoanodes. The transient decay time in the photoanodes was calculated from the logarithmic plot of parameter *D*, given by Eq. (3):

3 $$D = \, \left( {I_{{\text{t}}} {-}I_{{\text{s}}} } \right)/\left( {I_{{\text{m}}} {-}I_{{\text{s}}} } \right)$$

where *I*_m_ is the photocurrent spike, *I*_t_ is the photocurrent at time *t*, and *I*_s_ is the steady-state photocurrent (i.e., as the recombination and charge generation reaches equilibrium). The transient decay time is defined as the time at which in ln *D* = -1. Based on the photocurrent profiles measured in Fig. 5a, the transient decay time of the TiO_2_ NFs, BiVO_4_/TiO_2_ NFs, TiO_2_ NRs, and BiVO_4_/TiO_2_ NRs is displayed in Fig. 5b. As shown in Table S2, the transient decay time for the BiVO_4_/TiO_2_ NFs was 26.35 s, which is approximately 4.15 times longer than that of the TiO_2_ NFs at about 6.36 s. It represents a lower charge recombination rate of BiVO_4_/TiO_2_ NFs compared to the TiO_2_ NFs. Otherwise, the BiVO_4_/TiO_2_ NRs showed a decay time of 1.57 s, which is shorter than that of the TiO_2_ NRs at about 2.58 s. It is also related to the poor charge separation between BiVO_4_ nanodots and TiO_2_ NRs. These results indicate that TiO_2_ NFs can play a key role as a hole blocking layer, which expedites electron transport and reduces charge recombination.

To gain a deeper insight into charge recombination behaviors according to the TiO_2_ facet, time-resolved photoluminescence (TRPL) analysis was carried out at a wavelength of 540 nm, as shown in Fig. 5c. TRPL is a powerful technique to gain information about dynamics of the photogenerated electron and hole pairs, reflecting the charge recombination/separation behavior and charge trapping process in defects [38–40]. The TRPL results were fitted with a biexponential decay function, and photoluminescence lifetimes were calculated as represented in Table S3. The nonradiative (*τ*_1_) and radiative recombination lifetimes (*τ*_2_) of the photoexcited electrons and holes can be determined by measuring the luminescence signals resulting from recombination [38, 40]. The nonradiative recombination lifetime is generally determined from the surface recombination by the trap sites. Since, in both heterostructured photoelectrodes, it is the same BiVO_4_ that is the part in contact with the electrolyte on the band structures, they have similar *τ*_*1*_ values. However, the electron injection behavior into adjacent layers can be investigated by monitoring the radiative recombination lifetime. If the photoexcited carriers can be effectively transported into the neighboring layers, the radiative recombination lifetime of the heterostructured photoelectrode decreases. In this regard, the smaller *τ*_*2*_ value of BiVO_4_/TiO_2_ NFs (5.20 ns) than that of BiVO_4_/TiO_2_ NRs (6.10 ns) denotes the efficient extraction of photoexcited electrons by TiO_2_ NFs. It corresponds with the transient decay time analysis in Fig. 5b. Based on the analysis of charge carrier dynamics, we demonstrated that the crystal facet engineering of TiO_2_ nanostructures is a powerful tool to allow TiO_2_ to be utilized as the hole blocking layer for BiVO_4_ by boosting charge separation efficiency.

**Fig. 6:**
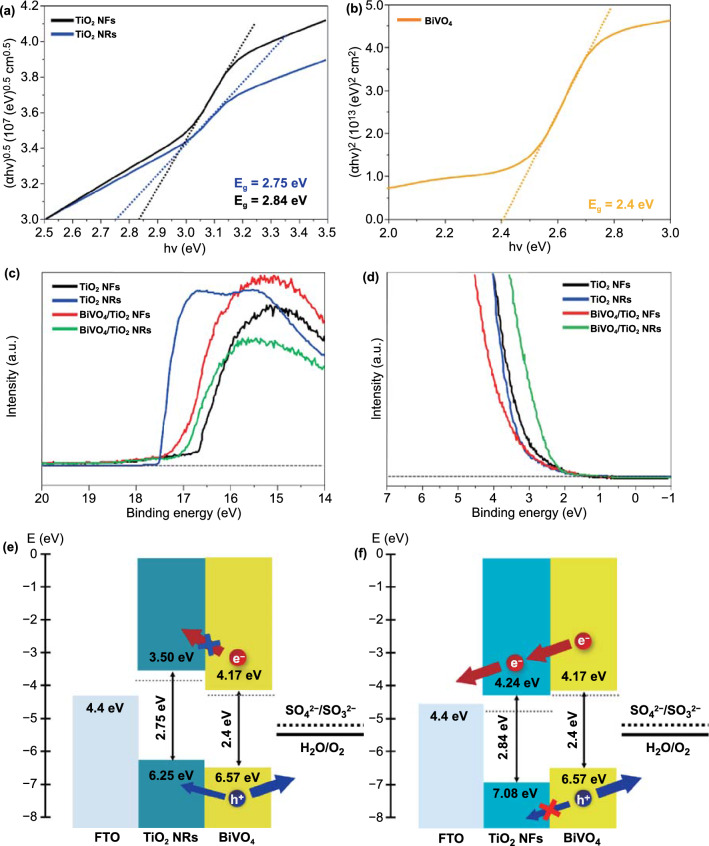
UV–Vis Tauc plots of **a** TiO_2_ NFs, TiO_2_ NRs, and **b** BiVO_4_. **c** Secondary electron emission spectra and **d** valence band spectra of TiO_2_ NFs, TiO_2_ NRs, BiVO_4_/TiO_2_ NFs, and BiVO_4_/TiO_2_ NRs. Flat band structures of **e** BiVO_4_/TiO_2_ NRs and **f** BiVO_4_/TiO_2_ NFs

To investigate the reason for the charge separation difference of TiO_2_ NRs with (001) facets and TiO_2_ NFs with (110) facets, we compared the band structures of BiVO_4_/TiO_2_ NRs and BiVO_4_/TiO_2_ NFs. The UPS measures occupied electronic states, providing information on the Fermi level and valence band maximum (VBM) energy of a material. The conduction band minimum (CBM) energy can be calculated by adding the optical band gap energy (*E*_g_), which is given by the UV–Vis spectroscopy, to the VBM. The band gap can be evaluated from Eq. (4):

4 $$\alpha \left( {hv} \right) = A\left( {hv \, - \, E_{g} } \right)^{n/2}$$

where *α*, *E*_g_, and *A* are the absorption coefficient, band gap energy, and a constant, respectively. Based on the UV–Vis spectra (Fig. S13) and Tauc plots, the optical band gap of the TiO_2_ NFs and TiO_2_ NRs was 2.84 and 2.75 eV, respectively (Fig. 6a). These are the smaller values than the band gap in previous reports (~ 3.2 eV). Because co-doped sulfur and nitrogen from the sulfamic acid form S 3p and N 2p state between the conduction band and valence band of TiO_2_, diminishing the band gap of TiO_2_ nanostructures [18]. And, a smaller band gap of TiO_2_ NRs compared to that of TiO_2_ NFs caused the absorption of the larger wavelength of visible light. Thus, as shown in Fig. 5a, the TiO_2_ NRs generated a higher photocurrent density at 1.23 V versus RHE than TiO_2_ NFs. According to Fig. 6b, the band gap of BiVO_4_ was 2.4 eV, which is similar to the values in previous studies.

The secondary electron emission (SEE) spectra of the TiO_2_ NFs, TiO_2_ NRs, BiVO_4_, BiVO_4_/TiO_2_ NFs, and BiVO_4_/TiO_2_ NRs were given by the high-binding-energy region in UPS, as shown in Fig. 6c. The work functions of TiO_2_ NFs (4.46 eV), TiO_2_ NRs (3.58 eV), BiVO_4_/TiO_2_ NFs (4.18 eV), and BiVO_4_/TiO_2_ NRs (4.18 eV) could be estimated from the differences between SEE spectra cutoffs and He I photon source energy (21.22 eV), as shown in Fig. S14. According to the valence band (VB) spectra given by the low-binding-energy region in UPS, the energy difference between the Fermi level and the valence band maximum (*E*_F_–*E*_VBM_) could be calculated, as shown in Fig. 6d. The values of *E*_F_–*E*_VBM_ for the TiO_2_ NFs (2.62 eV), TiO_2_ NRs (2.67 eV), BiVO_4_/TiO_2_ NFs (2.39 eV), and BiVO_4_/TiO_2_ NRs (2.39 eV) were calculated by the VB spectra cutoffs, as shown in Fig. S15. The values measured by UPS are summarized in Table S4. Since the band edge positions are highly dependent on the change of electrostatic potential in near-surface atoms, the VBM and CBM shifts between TiO_2_ NRs and TiO_2_ NFs is derived from the preferential crystal facet [41–43]. The titanium atoms on the (001) surface are tetrahedral-coordinated, and thus their environment is relatively close to the bulk. It causes the splitting of the occupied and unoccupied levels to be large, which leads to the highest CBM value [36, 37, 41]. Therefore, the (110) facet-dominant TiO_2_ NFs have a more negative CBM than the (001) facet-dominant TiO_2_ NRs. Based on these results, the energy band diagrams for the BiVO_4_/TiO_2_ NRs and BiVO_4_/TiO_2_ NFs are illustrated in Fig. 6e, f, respectively. In the case of BiVO_4_/TiO_2_ NRs, the CBM of the TiO_2_ NRs significantly move upward, making the transport of photogenerated electrons from BiVO_4_ difficult [38, 40, 44, 45]. Also, due to the relatively positive VBM of the TiO_2_ NRs, photogenerated holes from BiVO_4_ cannot be effectively blocked, causing the charge recombination, whereas TiO_2_ NFs having a relatively negative CBM and VBM can be effective electron transport layer and hole-blocking layer for BiVO_4_. As a result, the BiVO_4_/TiO_2_ NFs clearly form the type II band alignment, which is energetically favorable to separate the photogenerated electrons and holes from BiVO_4_. In the final analysis, it was found that the enhanced photoactivity of BiVO_4_/TiO_2_ NFs was originated from the improvement of charge separation according to the band structure of crystal facet-engineered TiO_2_.

## Conclusions

In summary, we have demonstrated the effect of crystal facet-controlled TiO_2_ nanostructures on band structures and charge separation. Two types of BiVO_4_/TiO_2_ heterostructure photoanodes having TiO_2_ NRs with (001) facets and TiO_2_ NFs with (110) were systematically studied to reveal the role of facets for facile PEC water splitting. We revealed that the improved photoelectrochemical performances of BiVO_4_/TiO_2_ NFs compared with BiVO_4_/TiO_2_ NRs were attributed to the prohibition of charge recombination through interface and morphology control. According to the analysis of charge carrier dynamics such as transient decay time and time-resolved photoluminescence, the charge separation effect was supported. Based on the UPS, it was represented that crystal facet engineering affects the band edge position of TiO_2_. In conclusion, this study not only provides new perspectives in band structure engineering but also opens a promising avenue in designing heterostructure photoelectrodes for efficient PEC water splitting.

## Supplementary Information

Below is the link to the electronic supplementary material.

Supplementary file1 (PDF 1261 KB)
