# Supplementary material for: Crystal Facet Engineering of TiO2 Nanostructures for Enhancing Photoelectrochemical Water Splitting with BiVO4 Nanodots
Source: Nanomicro Lett. 2022 Jan 25;14:48. doi: 10.1007/s40820-022-00795-8 (PMC8789981; doi:10.1007/s40820-022-00795-8)
Supplement: Supplementary file 1 — Supplementary file1 (PDF 1261 KB) [file 40820_2022_795_MOESM1_ESM.pdf]

Supporting Information for

Crystal Facet Engineering of TiO_2 Nanostructures for Enhancing Photoelectrochemical Water Splitting with $BiVO_4$ Nanodots

Mi Gyoung Lee[1, †], Jin Wook Yang[1, †], Hoonkee Park[1], Cheon Woo Moon[2], Dinsefa M. Andoshe[1], Jongseong Park[1], Chang-Ki Moon[1], Tae Hyung Lee[1], Kyoung Soon Choi[3], Woo Seok Cheon[1], Jang-Joo Kim[1], and Ho Won Jang[1, 4, *]

[1]Department of Materials Science and Engineering, Research Institute of Advanced Materials, Seoul National University, Seoul 08826, Republic of Korea

[2]Department of Chemistry and Nanoscience, Ewha Womans University, Seoul 03693, Republic of Korea

[3]National research Facilities & Equipment Center, Korea Basic Science Institute, Daejeon 34133, Republic of Korea

[4]Advanced Institute of Convergence Technology, Seoul National University, Suwon 16229, Republic of Korea

[†]M. G. L. and J. W. Y. contributed equally to this work

*Corresponding author. E-mail: hwjang@snu.ac.kr (Ho Won Jang)

Supplementary Tables and Figures

Table S1 Equivalent circuit analysis of EIS spectra for TiO_2 NFs, TiO_2 NRs, $BiVO_4/TiO_2$ NFs, and $BiVO_4/TiO_2$ NRs with different deposition cycles of $BiVO_4$

Photoanodes	R_s (Ω cm^2)	R_{ct1} (Ω cm^2)	R_{ct2} (Ω cm^2)
TiO_2 NFs	4.99	3.16	4770.75
6 $BiVO_4/TiO_2$ NFs	2.12	1.05	588.16
12 $BiVO_4/TiO_2$ NFs	3.26	16.68	599.46
18 $BiVO_4/TiO_2$ NFs	2.67	0.84	475.11
24 $BiVO_4/TiO_2$ NFs	2.41	1.01	743.04
36 $BiVO_4/TiO_2$ NFs	3.33	1.62	743.04
TiO_2 NRs	2.11	2.06	259.89
$BiVO_4/TiO_2$ NRs	3.34	5.18	1948.06

Table S2 Transient decay times of TiO_2 NFs, TiO_2 NRs, $BiVO_4/TiO_2$ NFs, and $BiVO_4/TiO_2$ NRs

Photoanodes	Transient decay time (s)
TiO_2 NFs	6.36
TiO_2 NRs	2.58
$BiVO_4/TiO_2$ NFs	26.35
$BiVO_4/TiO_2$ NRs	1.57

Table S3 TRPL lifetimes of TiO_2 NFs, TiO_2 NRs, $BiVO_4/TiO_2$ NFs, and $BiVO_4/TiO_2$ NRs

Photoanodes	τ_1 (ns)	τ_2 (ns)
TiO_2 NFs	0.7397	6.437
TiO_2 NRs	0.5978	4.314
$BiVO_4/TiO_2$ NFs	0.8009	5.2046
$BiVO_4/TiO_2$ NRs	0.7577	6.102

Table S4 a Energy level of valence band and work function of FTO, TiO_2 NFs, TiO_2 NRs, $BiVO_4/TiO_2$ NFs, and $BiVO_4/TiO_2$ NRs investigated by ultraviolet photoemission spectroscopy. **b** Optical band gap of TiO_2 NFs, TiO_2 NRs, and $BiVO_4$ measured by UV-visible spectroscopy

a

Materials	E_F-E_{VBM} (eV)	E_F (eV)
FTO	N/A	4.4
TiO_2 NFs	2.62	4.46
TiO_2 NRs	2.67	3.58
$BiVO_4/TiO_2$ NFs	2.39	4.18
$BiVO_4/TiO_2$ NRs	2.39	4.18

b

Materials	Optical band gap (eV)
FTO	N/A
TiO_2 NFs	2.84
TiO_2 NRs	2.75
$BiVO_4$	2.4

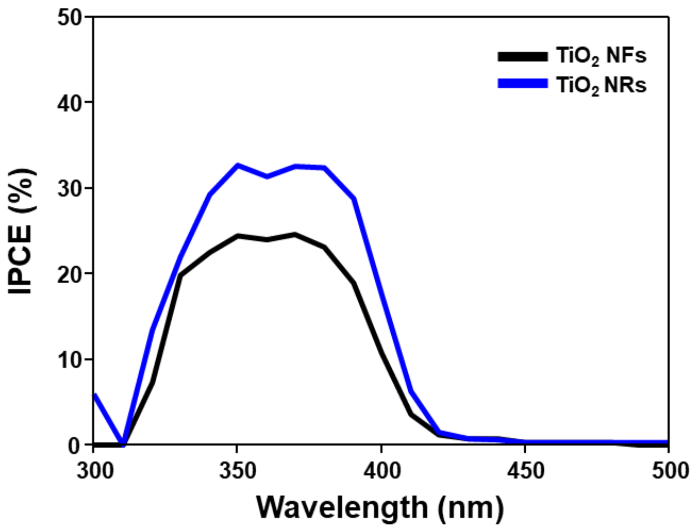

Fig. S1 IPCE at 1.23 V (vs. RHE) of TiO_2 NFs and TiO_2 NRs

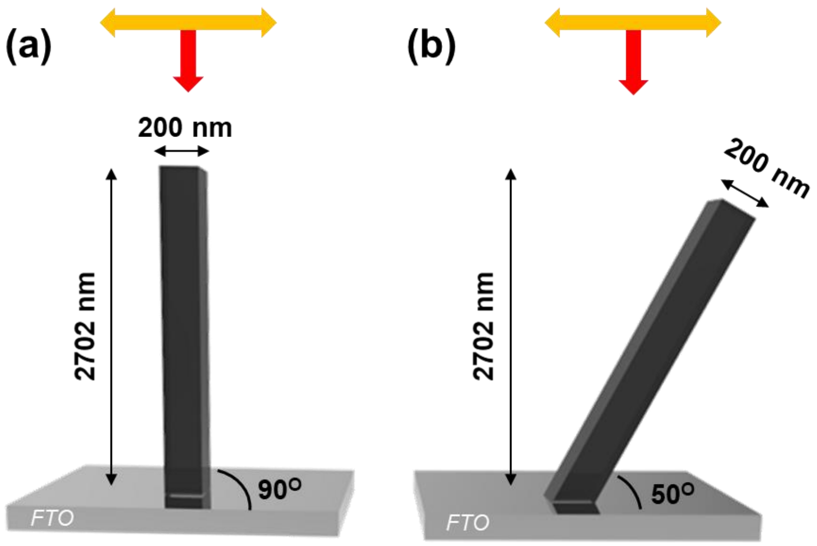

Fig. S2 Model used in finite-difference time-domain (FDTD) simulations of **a** TiO_2 NRs and **b** TiO_2 NFs

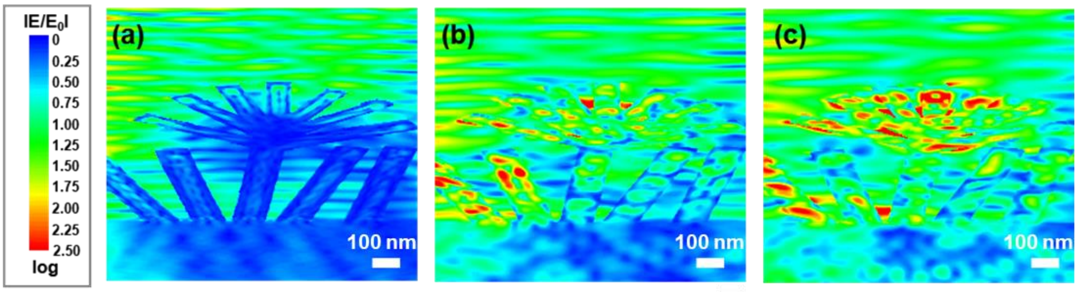

Fig. S3 FDTD simulations of near field enhancement of TiO_2 NFs on FTO under **a** 350 nm, **b** 450 nm, **c** 550 nm photons

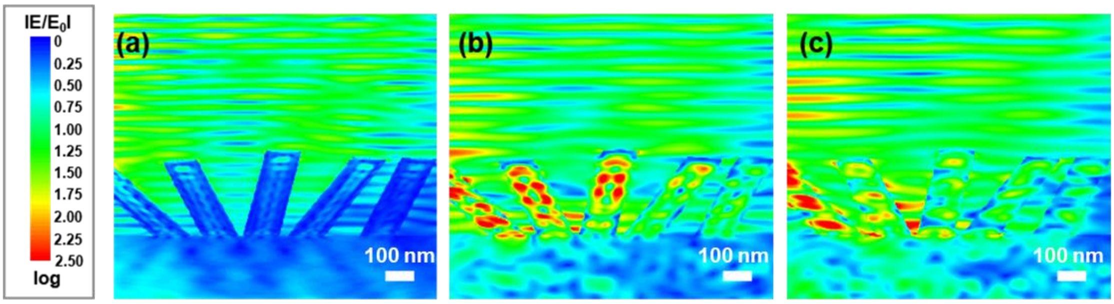

Fig. S4 FDTD simulations of near field enhancement of TiO_2 NFs on FTO under **a** 350 nm, **b** 450 nm, **c** 550 nm photons

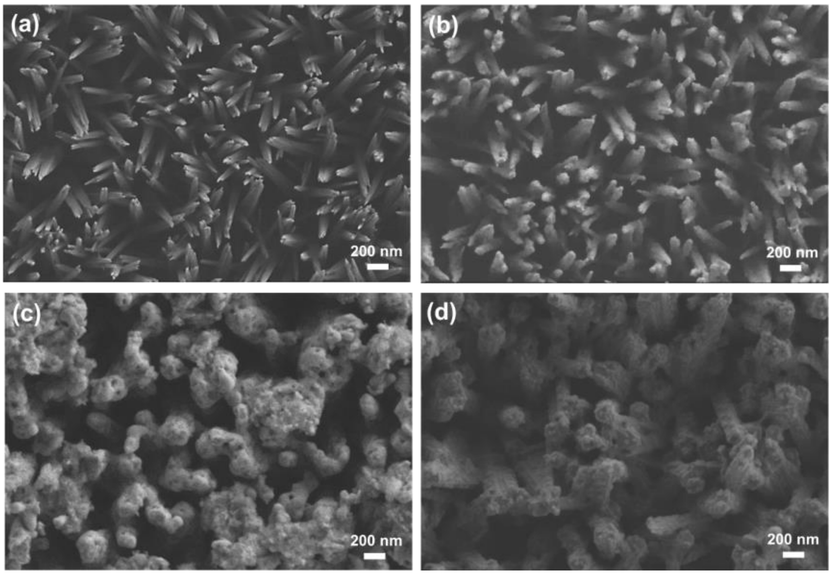

Fig. S5 SEM images of $BiVO_4/TiO_2$ NRs with different deposition cycles of $BiVO_4$: **a** 6 cycles, **b** 12 cycles, **c** 18 cycles, and **d** 36 cycles

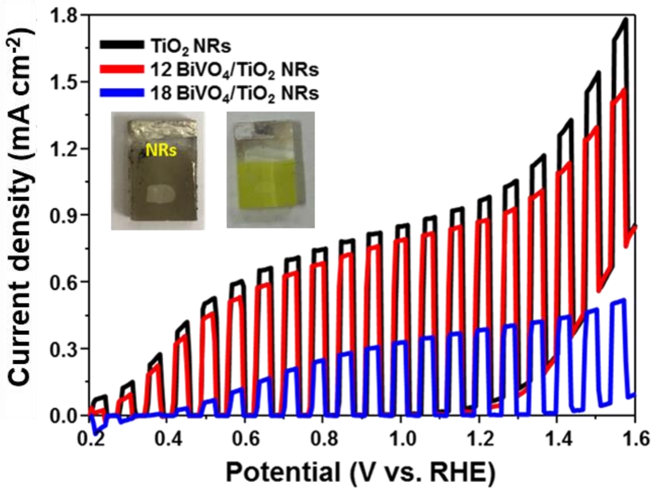

Fig. S6 LSVs of TiO_2 NRs and $BiVO_4/TiO_2$ NRs with different deposition cycles of $BiVO_4$ in 0.5 M K-P$_i$ buffer with 1 M Na_2SO_3

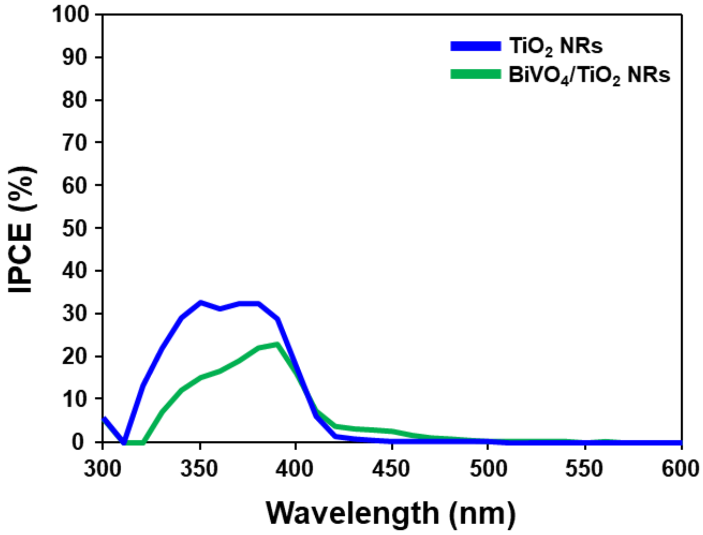

Fig. S7 IPCE at 1.23 V (vs. RHE) of TiO_2 NRs and $BiVO_4/TiO_2$ NRs

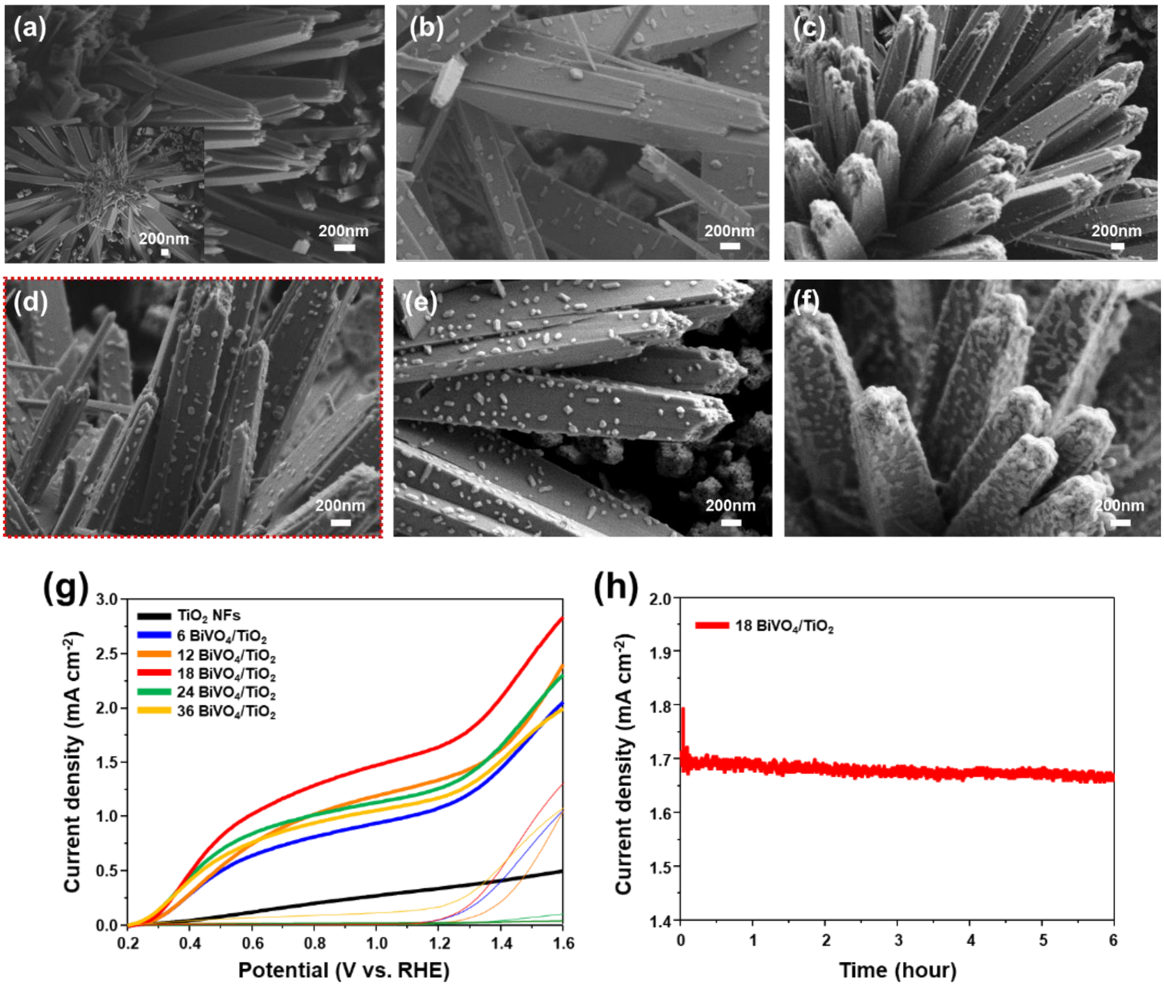

Fig. S8 SEM images of **a** TiO_2 NFs and $BiVO_4/TiO_2$ NFs with different deposition cycles of $BiVO_4$: **b** 6 cycles, **c** 12 cycles, **d** 18 cycles, **e** 24 cycles, and **f** 36 cycles. **g** LSV of TiO_2 NFs and $BiVO_4/TiO_2$ NFs with different deposition cycles of $BiVO_4$ in 0.5 M K-P_i buffer with 1 M Na_2SO_3. **h** Chronoamperometry of $BiVO_4/TiO_2$ NFs deposited by 18 cycles

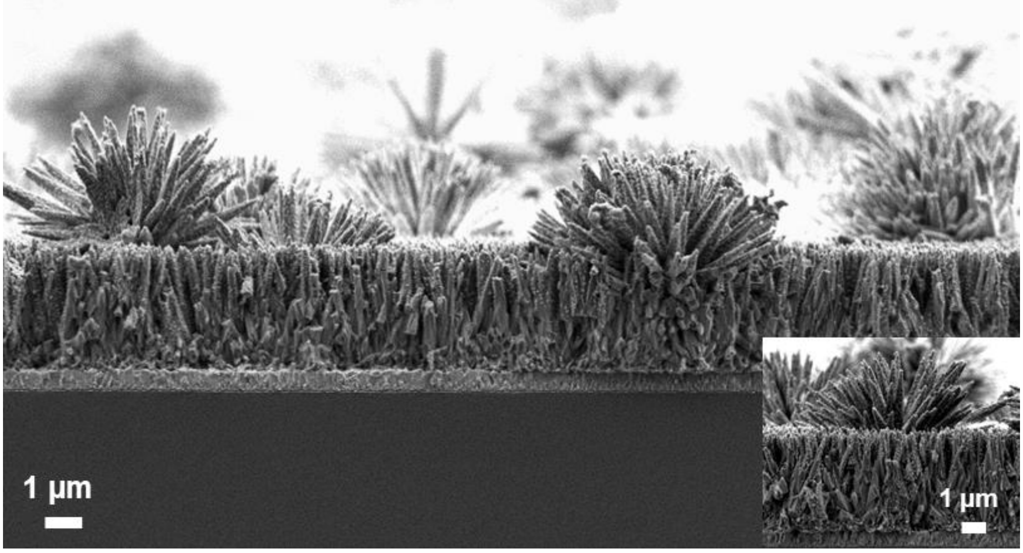

Fig. S9 Cross-sectional SEM images of $BiVO_4/TiO_2$ NFs deposited by 18 cycles. Inset is the enlarged image of $BiVO_4/TiO_2$ NFs

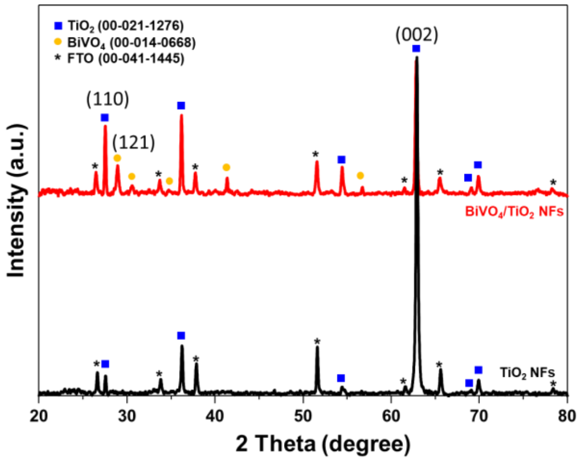

Fig. S10 XRD of TiO_2 NFs and $BiVO_4/TiO_2$ NFs

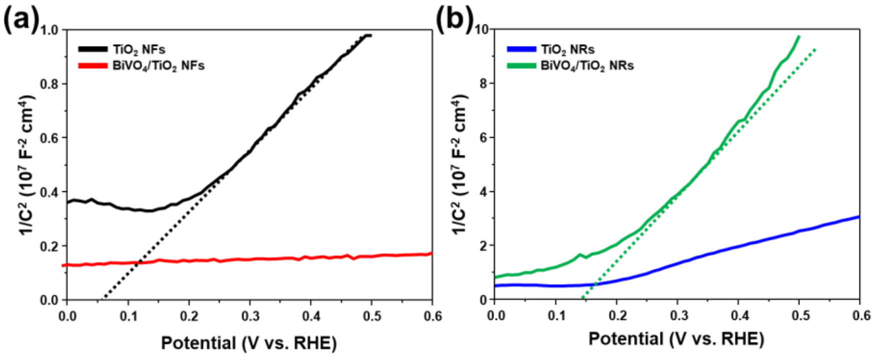

Fig. S11 Comparison of Mott-Schottky plot before and after deposition of $BiVO_4$ nanodots on the **a** TiO_2 NFs and **b** TiO_2 NRs. (Frequency: 1 kHz, Amplitude: 10 mV, under light off)

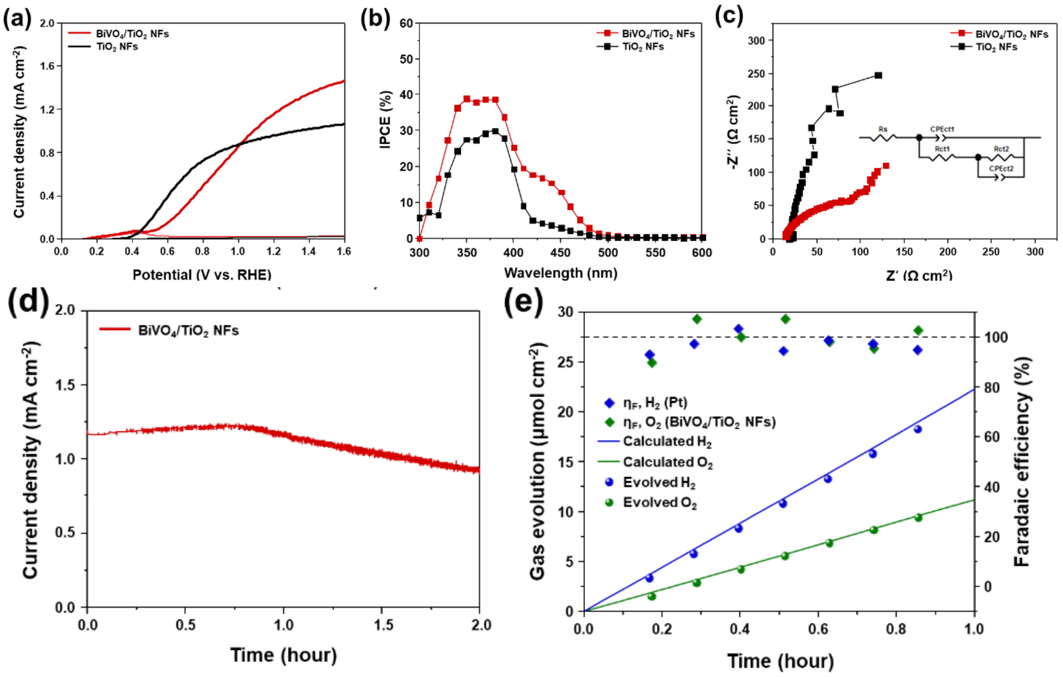

Fig. S12 a LSV (water oxidation) of TiO_2 NFs and 18 $BiVO_4/TiO_2$ NFs in 0.5 M K-P_i buffer without Na_2SO_3. **b** IPCE and **c** EIS of TiO_2 NFs and 18 $BiVO_4/TiO_2$ NFs at 1.23 V (vs. RHE) in 0.5 M K-P_i buffer without Na_2SO_3. **d** Chronoamperometry of 18 $BiVO_4/TiO_2$ NFs photoanode in 0.5 M K-P_i buffer without Na_2SO_3. **e** Gas evolution and Faradaic efficiency of 18 $BiVO_4/TiO_2$ NFs photoanode

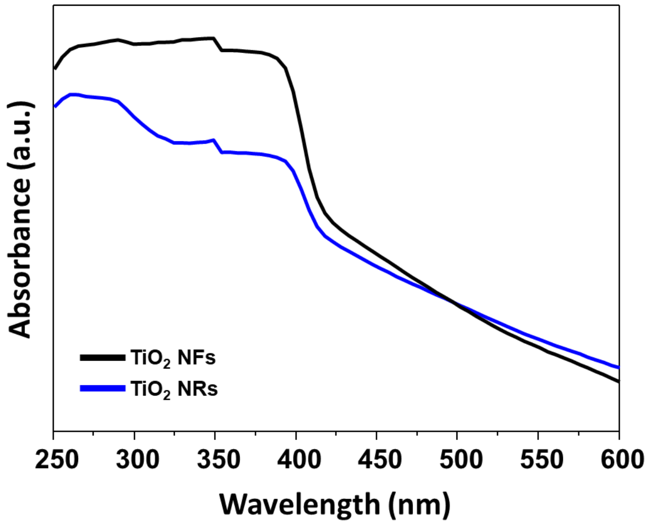

Fig. S13 UV-vis absorption spectra of TiO_2 NFs and TiO_2 NRs

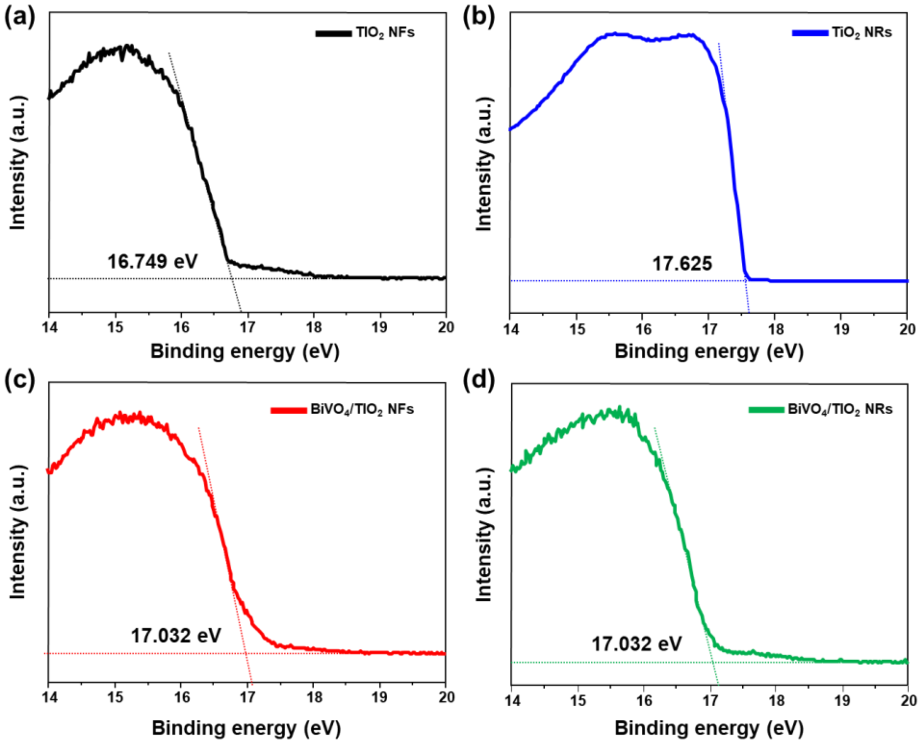

Fig. S14 Secondary electron emission spectra of **a** TiO_2 NFs, **b** TiO_2 NRs **c** $BiVO_4/TiO_2$ NFs, and **d** $BiVO_4/TiO_2$. $E_F = E_{cut\ off}$ - 21.21 eV (He I source)

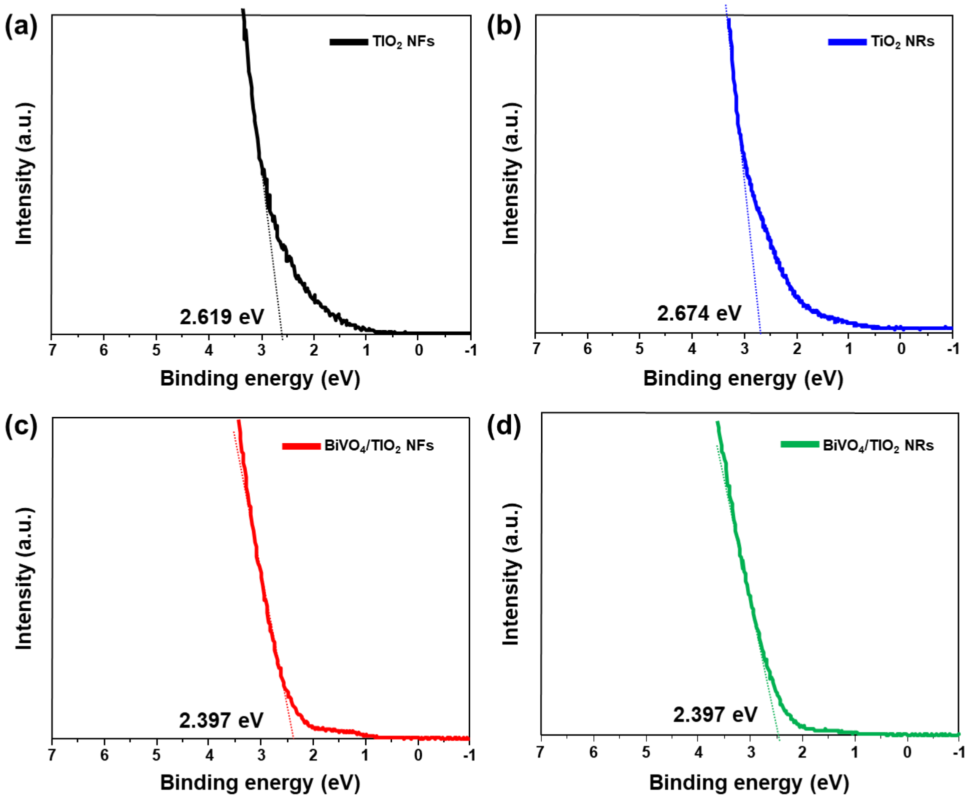

Fig. S15 Valence band spectra and the energy difference between Fermi level and valence band maximum (E_F - E_{VBM}) of **a** TiO_2 NFs, **b** TiO_2 NRs, **c** $BiVO_4/TiO_2$ NFs, and **d** $BiVO_4/TiO_2$ NRs. $E_{VBM} = E_F + E_{edge}$
